# Association between serum phosphate levels and length of hospital stay in infants with neonatal sepsis: a retrospective cohort study

**DOI:** 10.1186/s12887-025-06209-z

**Published:** 2025-10-21

**Authors:** Zhifang Du, Qingsheng Huang, Lisha Bao, Dan Li, Jing Li

**Affiliations:** 1 Neonatal Department, Bethune International Peace Hospital, 050082 Shijiazhuang, China; 2 Department of Critical Care Medicine, Third Hospital of HeBei Medical University , Shijiazhuang, HeBei 050051 China

**Keywords:** Phosphate, Neonatal sepsis, Outcome, Hypophosphatemia, Hyperphosphatemia, In-hospital length of stay

## Abstract

**Background:**

Research on the association of serum phosphate levels with the severity and prognosis of neonatal diseases is limited. Neonatal sepsis is the primary cause of neonatal mortality. Therefore, in this study, we aimed to investigate the association between serum phosphate levels and neonatal sepsis outcomes.

**Methods:**

This retrospective cohort study was conducted using the Pediatric Intensive Care (PIC) database (2010–2018). Neonatal sepsis was diagnosed based on ICD-10 codes. Serum phosphate levels within 72 h of sepsis diagnosis were selected. Outcomes were severe sepsis, in-hospital mortality, length of hospital stay (hospital Los.), and ICU stay (ICU Los.). Covariates included demographic and clinical characteristics as well as serum biomarkers. Multi-variable regression and subgroup analyses were performed to explore the association between serum phosphate levels and neonatal sepsis outcomes.

**Results:**

A total of 120 infants with neonatal sepsis were included, and their characteristics were analyzed according to serum phosphate tertiles. The median age was 3.0 (1.0, 17.0) days. The proportion of male infants was 62.5% (75/120). The proportion of preterm infants was 44.2% (53/120). The incidence of late-onset sepsis was 60.8% (73/120). Multivariate linear regression analysis showed that the serum phosphate level at sepsis diagnosis was associated with hospital Los. and remained so after adjustment for all covariates. Each 1 mmol/L decrease in serum phosphate level prolonged the length of hospital stay by 7.53 days. The low serum phosphate group had an 11.75-day longer hospital stay than the high serum phosphate group. Curve fitting showed a negative linear correlation between serum phosphate levels and hospital Los. in infants with neonatal sepsis and no significant interactions were observed in the subgroup analysis; however, serum phosphate levels were not independently associated with ICU Los. In addition, no correlation was found between serum phosphate levels and mortality or the severity of sepsis in neonates.

**Conclusions:**

Hypophosphatemia is highly prevalent in neonates with sepsis. Lower serum phosphate levels are associated with prolonged hospital stay in neonates with sepsis. Strengthening the monitoring of serum phosphate levels in neonates with sepsis and maintaining the homeostasis of phosphate in the body can promote refined management of neonatal sepsis and help improve the prognosis of the patients.

**Supplementary Information:**

The online version contains supplementary material available at 10.1186/s12887-025-06209-z.

## Introduction

Neonatal sepsis is a systemic inflammatory response syndrome in infants resulting from infection with various neonatal pathogens; its prevalence is 4.5%–9.7% of surviving newborns and mortality is between 11% and 19% [1]. In neonatal sepsis, the key measures to reduce mortality and improve outcomes include early detection and treatment, as well as meticulous management. Currently, research has focused on the predictors of neonatal sepsis, which include inflammatory and infectious biomarkers such as C-reactive protein, procalcitonin (PCT), leukocyte count, platelet count, and interleukin 6 [2, 3]. However, studies on the correlation of other potential biomarkers with the severity and outcomes of neonatal sepsis are limited.

Phosphorus is a multivalent ion critical for several physiological functions. Intracellular phosphorus is essential for signaling, nucleic acid synthesis, membrane function, energy metabolism, and other cellular processes. Phosphorus is a component of erythrocyte 2,3-diphosphoglycerate (2,3-DPG), which is involved in oxygen transport. Phosphate is also involved in maintaining the pH of plasma and urine [4]. Patients with sepsis can develop phosphate metabolism disorders. However, the relationship between serum phosphate levels and outcomes in patients with sepsis remains controversial [5–7].

Phosphorus metabolism in neonates differs from that of adults and children. Juvenile animals must maintain a positive phosphate balance to ensure maximal bone mineralization. The highest serum phosphate concentration is observed in infants and it decreases with age to typical adult values [8]. However, the overall incidence of hypophosphatemia is more common (88%) in neonatal sepsis [9]. Due to the important role of phosphorus in maintaining normal physiological functions in newborns, phosphate metabolism disorders can affect cellular energy metabolism, leading to organ dysfunction, and hypophosphatemia can inhibit neutrophil function. Therefore, phosphate metabolism disorders may affect the prognosis of neonatal sepsis.Studies on serum phosphate changes in neonatal sepsis remain limited. The direction of early phosphate derangement (high or low) and its predictive ability for prognosis in neonatal sepsis remains unclear. Therefore, in this study, we assessed the association of the serum phosphate levels (within 72 h of diagnosing sepsis) with outcome in infants with sepsis.

## Materials and methods

### Database

In this retrospective observational cohort study, we used data from the Pediatric Intensive Care (PIC) database. This is a large, open, specialized, single-center pediatric database [10]. The database that contains comprises information on 12,881 pediatric patients with 13,941 hospitalizations in various ICUs at the Children’s Hospital, Zhejiang University School of Medicine, China, from 2010 to 2018. This study was approved by The project obtained approval from the Institutional Review Board of Children’s Hospital, Zhejiang University School of Medicine, Hangzhou, China. The study protocol was approved by the Medical Ethics Committee of the Bethune International Peace Hospital(2025-KY-115).As this study did not involve a clinical intervention, informed patient consent was not required.

### Study population

Patients with neonatal sepsis were included in this study. The diagnosis of neonatal sepsis was based on the ICD-10 code in the discharge diagnosis, indicating sepsis or neonatal sepsis, and fulfilled the diagnostic criteria of International Pediatric Consensus Conference statement on sepsis and organ dysfunction in pediatrics [11, 12]. Premature birth was diagnosed using ICD-10 codes. Inclusion criteria were as follows: Patients who met the criteria for neonatal sepsis (1) preterm infants aged ≤ 90 days, term infants aged ≤ 28 days; (2) first ICU admission; and infants with data on serum phosphate testing. Patients who lacked data on serum phosphate levels within 72 h of diagnosing sepsis were excluded. No restrictions were placed on the time period of in-hospital mortality.

### Exposure variable

The closest available serum phosphate recording within 72 h after the diagnosis of sepsis (based on the time of collection of the positive blood culture or indicators of non-specific infections) was extracted.

### Data extraction and covariates

Based on the literature, the following covariates were included: age, sex, ICU weight (closest available weight after ICU admission), preterm or term birth, in-hospital mortality, length of hospital stay (hospital Los.), length of ICU stay (ICU Los.), severe sepsis, late-onset sepsis (LOS), comorbidities including bacterial meningitis, digestive tract anomaly, necrotizing enterocolitis (NEC) and peritonitis, congenital heart disease (CHD), intracranial hemorrhage (ICH), neonatal asphyxia, acute kidney injury (AKI), respiratory failure, and acute liver injury (ALI). Laboratory tests included blood culture, blood pH, leukocyte count, platelet count, hemoglobin levels, and serum levels of ionized calcium (iCa), total calcium, alanine aminotransferase (ALT), blood urea nitrogen (BUN), creatinine (CR), C-reactive protein (CRP), and lactate (LAC). The most recent values within 72 h of diagnosing sepsis were used as variables. Severe sepsis was defined as sepsis in addition to one of the following conditions: cardiovascular organ dysfunction, acute respiratory distress syndrome, and dysfunction of two or more other organs [11]. LOS was defined as the onset of sepsis after 72 h of life [12]. Comorbidities were diagnosed based on ICD-10 codes. The diagnostic criteria for acute kidney injury (AKI) are serum creatinine ≥ 26.5 mmol/L or 150%–199% of the basal value [13]. ALI was defined as ALT levels >2 times the upper limit of normal for age [14]. Covariates were excluded in cases with more than 40% missing values. Multiple imputations were used to handle missing data, and model estimates and standard errors were calculated using Rubin’s rules.

### Outcomes

The outcomes included in-hospital mortality, severe sepsis, hospital Los. and ICU Los.

### Statistical analysis

Patient characteristics were analyzed according to two grouping methods (phosphate level tertiles and cut-off value of phosphate level at 2.0 and 2.6 mmol/L). Continuous variable data are presented as mean ± standard deviation or median with interquartile range, while categorical variables are presented as frequencies and percentages. Statistical analyses were performed to compare differences among the three groups and included the chi-squared or Fisher’s exact tests for categorical variables, one-way analysis of variance for normally distributed variables, and the Kruskal–Wallis H test for skewed distributions.

Uni-variate linear or logistic regression analyses were performed to evaluate the association between covariates and outcomes in infants with sepsis. Multivariate logistic regression analyses were performed to evaluate the association among serum phosphate levels, in-hospital mortality, and severe sepsis. Multivariate linear regression analyses were performed to evaluate the association between serum phosphate levels and hospital Los. We entered variables that were considered clinically relevant or all significant covariates (*P* < 0.1) into the uni-variate analysis. According to the recommendations of the Strengthening the Reporting of Observational Studies in Epidemiology (STROBE) statement [15], the analyses were first performed without adjustment. Further multivariate linear regression analyses for hospital Los. cumulatively included adjustments for age, sex, ICU weight, LOS, premature birth, hospital mortality, severe sepsis, CHD, respiratory failure, blood hemoglobin levels, and serum levels of CRP, iCa, CR, LAC, and ALT. Adjustment variables incorporated in multi-variable linear regression analyses for ICU Los. include age, sex, ICU weight, premature birth, LOS, mortality, severe sepsis, digestive tract anomaly, respiratory failure, blood pH, and serum levels of CRP and iCa. Adjustment variables incorporated in multi-variable logistic regression analysis models for in- hospital mortality and severe sepsis include age, sex, and ICU weight.

Smooth curve fitting was employed to assess the relationship between serum phosphate levels and hospital Los. in septic infants, and interaction and subgroup analyses were performed according to sex, premature birth, severe sepsis, LOS, AKI, and hypocalcemia using linear regression models.

All analyses were conducted using the R3.3.2 software package (http://www.R-project.org, R Foundation) and Free Statistics software version 1.8. Two-tailed tests were performed, and a *P*-value < 0.05 was considered statistically significant.

### Sensitivity analysis

For sensitivity analysis, we excluded premature infants aged > 28days and infants with missing variables.

## Results

### Participants selection

**Fig. 1:**
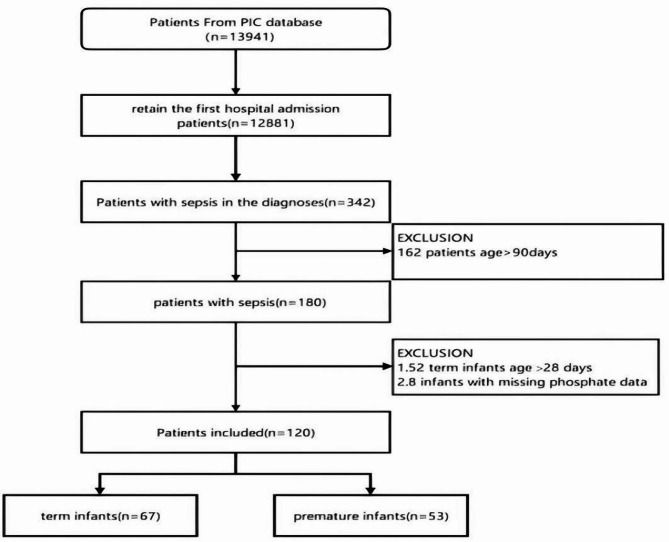
Flowchart of patient selection

Of the 13,941 records in the PIC database, 1,060 duplicates were excluded. Among the remaining 12,881 hospitalized patients, 342 with sepsis were identified (Fig. 1), and 120 patients with complete laboratory data were included (Fig. 1).

### Baseline characteristics

The baseline characteristics of the patients are presented in Table 1. Patients were grouped by the tertiles of serum phosphate level as follows: low group, ≥ 0.55 and ≤ 1.60 mmol/L; middle group, ≥ 1.61 and ≤ 2.12 mmol/L; and high group, ≥ 2.13 and ≤ 4.93mmol/L. The lowest and highest serum phosphorus levels in the study population were 0.55 mmol/L and 4.93 mmol/L, respectively.The mean age of all participants was 3 (1.0–17.0) days, and 75% were male. Preterm infants accounted for 44.2% of the cases, and 60.8% had late-onset sepsis. The overall in-hospital mortality was 8.3%. Severe sepsis accounted for 28.3% of all cases. We observed some differences between the different serum phosphate level groups with respect to various covariates (age, LOS, hospital Los., ICU Los., AKI, blood hemoglobin levels, and serum levels of iCa, CR, and LAC). The baseline characteristics of participants were also stratified by serum phosphate level at 2.0 and 2.6 mmol/L and presented in supplemental table S1. The percentages of infants with hypophosphatemia (< 2.0 mmol/L), hyperphosphatemia (> 2.6 mmol/L), and normal phosphatemia were 60% (72/120), 15.8% (19/120), and 24.1% (29/120), respectively. The comparison of baseline characteristics between the three groups was consistent with the results of the serum phosphate level tertials, except for iCa, which did not differ significantly among the three groups.Comparing baseline characteristics of infants with hypophosphatemia (< 2.0 mmol/L) grouped by serum phosphate level tertiles, no significant differences were found in various covariates, as shown supplemental table S9.

**Table 1:** Baseline characteristics of participants

Co variate	Total (*n* = 120)	Phosphate level(mmol/L)(mg/dl)			*P* -value
		Low(0.55–1.60)(1.71–4.96) *n* = 40	Mild(1.61–2.12)(4.99–6.57) *n* = 40	High(2.13–4.93)(6.6-15.28) *n* = 40	
Baseline characteristics					
age, days	3.0 (1.0, 17.0)	5.0 (2.0,27.2)	10.0 (2.0, 20.5)	2.0 (0.8, 4.0)	0.001
gender, Males, *n* (%)	75 (62.5)	23 (57.5)	29 (72.5)	23 (57.5)	0.278
ICU weight, kg	2.5 ± 0.9	2.4 ± 0.9	2.3 ± 1.0	2.6 ± 0.7	0.441
premature, *n* (%)	53 (44.2)	18(45)	22(55)	13(32.5)	0.127
LOS, *n* (%)	73 (60.8)	27 (67.5)	32(80)	14(35)	< 0.001
Outcome					
In-hospital Mortality, *n* (%)	10 (8.3)	2 (5.0)	2 (5.0)	6 (15)	0.209
severe.sepsis, *n* (%)	34 (28.3)	10 (25)	13 (32.5)	11 (27.5)	0.75
hospital.Los, days	28.9 ± 21.7	35.2 ± 23.5	33.4 ± 20.9	18.0 ± 16.1	< 0.001
ICU Los, days	24.8 ± 21.3	31.2 ± 24.4	28.2 ± 22.5	15.2 ± 11.7	0.001
Comorbidities					
bacterial meningitis, *n* (%)	13 (10.8)	4(10)	4 (10)	5 (12.5)	1.00
digestive tract anomaly, *n* (%)	13 (10.8)	5 (12.5)	5 (12.5)	3(7.5)	0.818
NEC and peritonitis, *n* (%)	11 (9.2)	5 (12.5)	4(10)	2 (5)	0.631
CHD, *n* (%)	15 (12.5)	5(12.5)	6(15)	4(10)	0.796
ICH, *n* (%),	21 (17.5)	7(17,5)	10(25)	4(10)	0.21
neonatal asphyxia, *n* (%)	9(7.5)	2 (5)	2 (5)	5(12.5)	0.49
respiratory failure, *n* (%)	23(19.2)	7 (17.5)	12 (30)	4(10)	0.072
AKI, *n* (%)	23 (19.2)	4(10)	6(15)	13(32.5)	0.027
ALI, *n* (%)	11 (9.2)	1 (2.5)	3 (7.5)	7(17.5)	0.091
Laboratory examination					
blood culture, positive, *n* (%)	47 (39.2)	18 (45)	15(37.5)	14 (35)	0.635
phosphate, mmol/L	1.9 ± 0.7	1.3 ± 0.2	1.9 ± 0.1	2.7 ± 0.6	< 0.001
iCa, mmol/L	1.1 ± 0.2	1.2 ± 0.2	1.1 ± 0.2	1.1 ± 0.2	0.019
Total Ca, mmol/L	2.1 ± 0.6	2.2 ± 1.0	2.1 ± 0.3	2.1 ± 0.3	0.831
ALT, U/L	14.0 (8.0, 28.2)	16.0 (9.0 38.5)	11.0 (7.8,17.5)	15.0 (9.0, 23.5)	0.338
BUN, mmol/L	5.8 ± 4.0	5.4 ± 3.2	5.4 ± 4.3	6.7 ± 4.2	0.218
CR, µmol/L	78.3 ± 40.5	69.0 ± 33.2	69.1 ± 3.87	96.9 ± 43.3	0.001
CRP, mg/L	52.6 ± 49.5	66.4 ± 61.6	47.5 ± 42.1	43.7 ± 39.5	0.398
WBC, ×10^9^/L	12.6 ± 10.2	14.3 ± 12.4	11.2 ± 8.9	12.3 ± 8.9	0.929
platelets, ×10^9^/L	175.5 ± 140.2	141.0 ± 121.3	197.0 ± 165.5	188.9 ± 126.2	0.156
Hemoglobin, g/L	131.9 ± 31.9	123.9 ± 31.2	127.7 ± 25.9	144.5 ± 35.1	0.009
PH	7.3 ± 0.3	7.3 ± 0.1	7.2 ± 0.4	7.3 ± 0.2	0.330
LAC, mmol/L	2.3(1.5,4.4)	2.0(1.5,2.6)	2.2(1.4,3.8)	3.0(2.0,6.6)	0.028

*Abbreviations*: *ICU* weight: first weight in ICU, *hospital.Los. *length of hospital stay, *ICU Los. *length of hospital stay, *LOS* Late onset neonatal sepsis, *NEC* necrotizing enterocolitis in newborns, *CHD* congenital heart disease, *ICH* intracranial hemorrhage, *AKI* acute renal injury, *ALI* acute liver function injury, *ALT* alanine aminotransferase, *BUN* blood urea nitrogen, *CRP* C reactive protein, *PH* pH value in blood gas, *LAC* lactic acid

### Association between serum phosphate levels and clinical outcomes

Uni-variate analysis indicated that [high/low] serum phosphate levels, [high/low] ICU weight, premature birth, LOS, in-hospital mortality, severe sepsis, presence of comorbid congenital heart disease and respiratory failure, as well as [high/low] serum levels of iCa, total Ca, and CR were associated with hospital Los. [High/low] serum phosphate levels, [high/low] ICU weight, premature birth, LOS, in-hospital mortality, severe sepsis, presence of comorbid respiratory failure, as well as [high/low] serum levels of iCa, total Ca, and CRP were associated with ICU Los.(Table 2). Serum phosphate levels did not correlate with mortality or severe sepsis. The results of the univariate analysis of covariates, in-hospital mortality, and severe sepsis are shown in supplemental tables S2 and S3.

**Table 2:** Association of covariates And in-hospital Los. And ICU Los

Variable	Hospital Los.		ICU Los.	
	β.(95%CI)	*P*-value	β.(95%CI)	*P*-value
Age(days)	0.22 (−0.02,0.46)	0.078	0.22 (−0.02,0.46)	0.074
Gender, *n* (%): Males	−1.05 (−9.17,7.07)	0.798	−2.51 (−10.49,5.47)	0.534
ICU Weight	−9.82 (−13.58,−6.06)	< 0.001	−11.81(−15.29,−8.32)	< 0.001
Premature, *n* (%):	19.08 (11.97,26.19)	< 0.001	16.5 (9.31,23.69)	< 0.001
LOS, *n* (%):	13.43 (5.76,21.11)	< 0.001	9.47(1.73,17.21)	0.017
Mortality, *n* (%)	−27.35 (−40.68,−14.03)	< 0.001	−23.12 (−36.47,−9.76)	< 0.001
Severe sepsis, *n* (%):	−13.74 (−22.1,−5.38)	0.001	−9.86(−18.26,−1.46)	0.022
Bacterial meningitis, *n* (%):	10.32 (−2.19,22.83)	0.105	3.14(−9.3,15.58)	0.618
Digestive tract anomaly, *n* (%)	−0.64 (−13.29,12.01)	0.921	−10.84 (−23.14,1.45)	0.083
CHD, *n* (%)	12.56 (0.9,24.23)	0.035	8.13 (−3.48,19.74)	0.168
NEC and peritonitis, *n* (%)	4.84 (−8.75,18.44)	0.482	−8.24(−21.57,5.09)	0.223
ICH, *n* (%)	4.94 (−5.37,15.25)	0.344	6.31(−3.81,16.43)	0.22
Neonatal asphyxia, *n*(%)	10.11(−4.71,24.92)	0.179	5.66(−9,20.31)	0.446
Respiratory failure, *n*(%)	11.02(1.24,20.8)	0.028	12.73(3.17,22.28)	0.009
ALI, *n*(%)	−5.77(−19.35,7.82)	0.402	−9.44(−22.74,3.86)	0.163
AKI, *n* (%)	−5.98 (−15.91,3.95)	0.236	−4.37 (−14.18,5.43)	0.379
Blood culture, positive, *n* (%)	4.61 (−3.4,12.62)	0.257	2.75 (−5.16,10.66)	0.493
Phosphate, mmol/L	−12.61 (−17.66,−6.65)	< 0.001	−10.59 (−16.11,−5.08)	< 0.001
iCa, mmol/L	34.52(11.56,57.47)	0.004	33.59(10.93,56.26)	0.004
Total Ca, mmol/L	7.88(1.9,13.86)	0.01	7.35(1.45,13.25)	0.015
CRP, mg/L	0.07 (−0.0,0.15)	0.065	0.08 (0.01,0.16)	0.036
WBC, ×10^9^/L	−0.03(−0.41,0.36)	0.895	−0.1(−0.48,0.28)	0.599
Hemoglobin, g/L	−0.07 (−0.19,0.06)	0.298	−0.04 (−0.16,0.08)	0.487
Platelets, ×10^9^/L	0.01(−0.02,0.04)	0.555	−0.02(−0.04,0.01)	0.277
ALT, U/L	0(−0.04,0.03)	0.947	0(−0.04,0.03)	0.841
BUN, mmol/L	−0.52(−1.51,0.47)	0.301	−0.53(−1.51,0.44)	0.282
CR,µmol/L	−0.1 (−0.19,0)	0.047	−0.06(−0.15,0.04)	0.251
PH	−9.53 (−24.28,5.21)	0.203	−13.43 (−27.87,1.01)	0.068
LAC, mmol/L	−0.81 (−1.67,0.05)	0.063	−0.56 (−1.41,0.29)	0.197

Multi-variable linear regression analyses were used to assess the association between serum phosphate levels and hospital Los. (Table 3). In unadjusted Model I, serum phosphate levels were negatively associated with hospital Los. (β, 12.16; 95% confidence interval [CI], − 17.61–−6.7; *P* < 0.001). Even after adjusting for all potential covariates, with serum phosphate levels expressed as continuous variable, in Model IV, the association remained significant (β, − 7.53; 95% CI, − 12.54–−2.51; *P* = 0.004), such that hospital Los. was prolonged by 7.53 days with each 1 mmol/L decrease in serum phosphate levels. When serum phosphate levels were entered in the fully adjusted model as a categorized variable, we found that patients with high phosphate levels exhibited a shorter hospital Los. of 11.75 days; the difference was statistically significant (β, − 11.75; 95% CI, − 19.82–−3.68; *P* = 0.005) compared with the low phosphate levels group in Model IV. However, we observed no significant difference hospital Los. between mild middle and low phosphate levels group in unadjusted Model I (β, − 1.8; 95% CI, − 10.73–7.13; *P* = 0.694) and adjusted Model IV (β, − 3.23; 95% CI, − 10.57–4.1; *P* = 0.389).

**Table 3:** Multi-variable linear regression analyses of phosphate level and hospital Los

	Phosphate(mmol/L)(mg/dl)		Phosphate tertiles					
	*n*=120		Low(0.55-1.60)(1.71-4.96)	Mild(1.61-2.12) (4.99-6.57)		High(2.13-4.93)(6.6-15.28)		*P* for trend
			*n*=40	*n*=40		*n*=40		
	β(95%CI)	*P*	Reference	β(95%CI)	*P*	β(95%CI)	*P*	
Model I	-12.16 (-17.61~-6.7)	<0.001	Reference	-1.8(-10.73~7.13)	0.694	-17.25(-26.18~-8.32)	<0.001	<0.001
Model Ⅱ	-11.61 (-17.27~-5.94)	<0.001	Reference	-1.52 (-10.57~7.54)	0.743	-16.41 (-25.68~-7.14)	0.001	0.001
Model III	-8.14 (-12.89~-3.38)	0.001	Reference	-3.48 (-10.81~3.86)	0.355	-13.06 (-20.78~-5.33)	0.001	0.002
Model IV	-7.53(-12.54~-2.51)	0.004	Reference	-3.23(-10.57~4.1)	0.389	-11.75(-19.82~-3.68)	0.005	0.006

Model I : no other covariates were adjusted

Model Ⅱ: we adjusted age and gender

ModelIII: adjusted as for model Ⅱ,additionally adjusted for ICU weight, premature,LOS,hospital mortality,severe sepsis,CHD,respiratory failure

ModelIV: adjusted as for model III,additionally adjusted for CRP, iCa, CR, LAC, ALT, hemoglobin

The correlation between serum phosphate level and ICU Los. was also explored using multivariate linear regression analysis (Table 4). In the unadjusted Model I, the correlation between serum phosphate levels and ICU Los. was statistically significant (β, − 10.59; 95% CI, − 16.06–−5.13; *P* < 0.001) and the high phosphate levels group had a shorter ICU Los. of 15.93 days (β, − 15.93; 95% CI, − 24.84–−7.02; *P* = 0.001) compared with the low phosphate levels group. However, after adjusting for all potential covariates in Model IV, the strong correlation between serum phosphate levels and ICU Los. became unstable (β, − 4.36; 95% CI, − 9.07–−0.36; *P* = 0.073); no significant difference was observed between the high and low phosphate level groups (β, − 7.6; 95% CI, − 15.35–−0.14; *P* = 0.057).

**Table 4:** Multi-variable linear regression analyses of phosphate level and ICU Los.

	Phosphate(mmol/L)(mg/dl)		Phosphate tertiles					
	*n* = 120		Low(0.55–1.60)(1.71–4.96)	Mild(1.61–2.12)(4.99–6.57)		High(2.13–4.93)(6.6-15.28)		*P* for trend
	β(95%CI)	*P*	Reference	β(95%CI)	*P*	β(95%CI)	*P*	
Model I	−10.59 (−16.06~−5.13)	< 0.001	Reference	−3.01(−11.92 ~ 5.9)	0.509	−15.93(−24.84~−7.02)	0.001	0.001
Model Ⅱ	−9.97 (−15.63~−4.31)	0.001	Reference	−2.55(−11.55 ~ 6.45)	0.58	−14.92 (−24.13~−5.7)	0.002	0.002
Model III	−6.12 (−10.99~−1.26)	0.015	Reference	−4 (−11.48 ~ 3.48)	0.297	−11.05(−18.94~−3.16)	0.007	0.008
Model IV	−4.36(−9.07 ~ 0.36)	0.073	Reference	−2.02(−9.262 ~ 5.22)	0.585	−7.6(-−15.35 ~ 0.14)	0.057	0.061

Model I : no other covariates were adjusted

Model Ⅱ: we adjusted age and gender

ModelIII: adjusted as for model Ⅱ,additionally adjusted for ICU weight, premature, LOS, hospital mortality, severe sepsis, digestive tract anomaly, respiratory failure

ModelIV: adjusted as for model III, additionally adjusted for CRP, iCa, PH

Multi-variable logistic regression analyses were performed to assess the associations among serum phosphate levels, in-hospital mortality, and severe sepsis (supplemental table S4). Serum phosphate levels was not associated with in-hospital mortality or severe sepsis neither as continuous nor as categorical variable before or after model adjustment.

### Linear relationship between serum phosphate levels and hospital Los

**Fig. 2:**
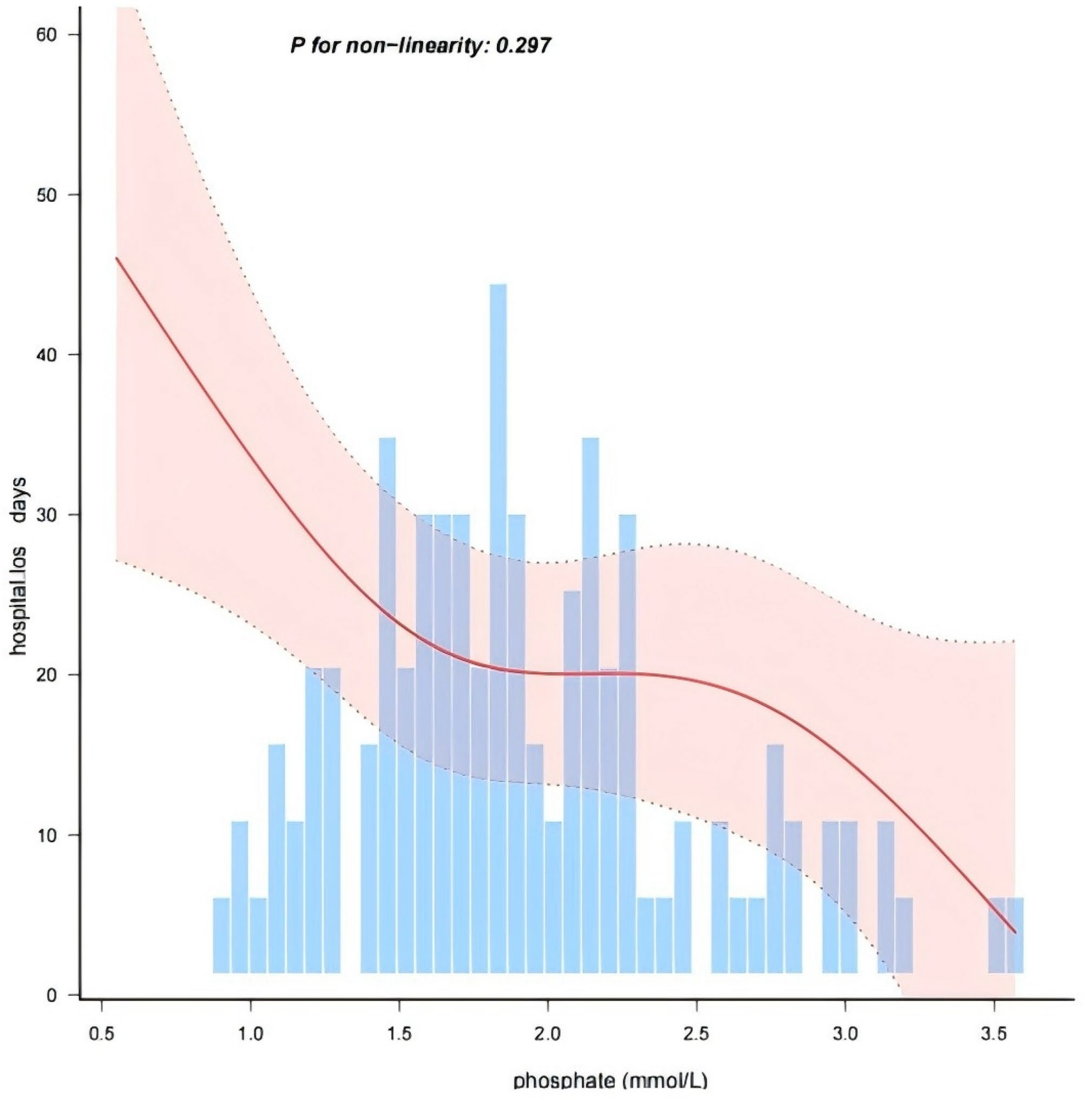
Linear dose-response relationship between phosphate level and length of hospital stay. Adjustment factors included age, gender, ICU weight, LOS, in hospital mortality, severe.sepsis, CHD, respiratoryfailure, CRP, iCa, ALT, LAC, hemoglobin, CR. The red line and pink area represent the estimated values and their corresponding 95% confidence intervals, respectively

We observed a negative linear dose-response relationship between serum phosphate levels and hospital Los. after adjusting for covariates (Fig. 2).

### Subgroup analysis

**Fig. 3:**
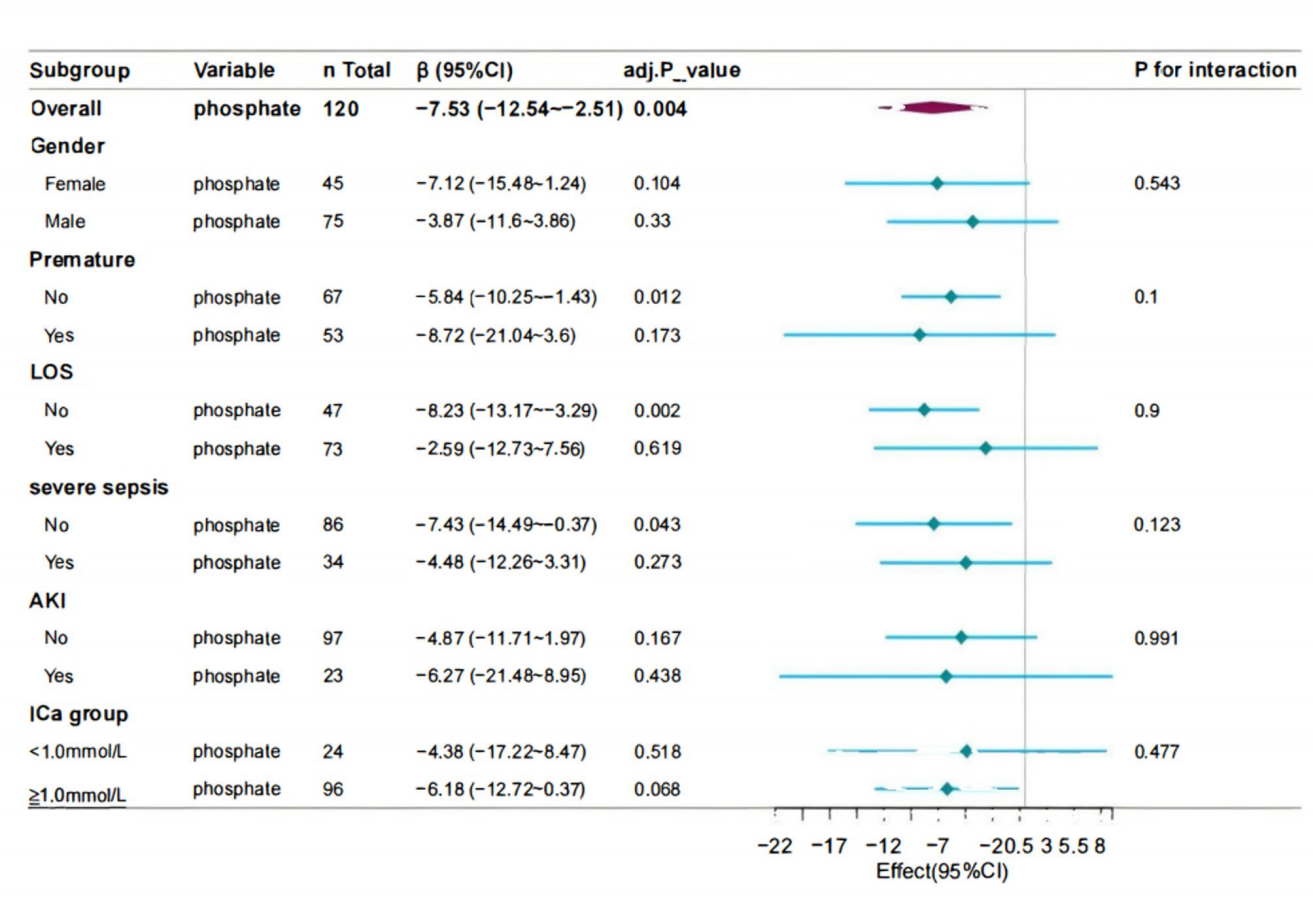
Subgroup analysis of the associations between phosphate level and the length of hospital stay

We conducted a subgroup analysis using gender, premature birth, LOS, severe sepsis, AKI, and hypocalcemia (iCa < 1.0 mmol/L) as stratification variables to explore the relationship between serum phosphate levels and hospital Los. (supplemental table S5). The analysis revealed no significant interaction between serum phosphate levels and the various subgroups (P-interaction > 0.05), indicating that the conclusions are stable and reliable across different subgroups (Fig. 3).

### Sensitivity analysis

In the sensitivity analysis, we excluded 14 premature infants aged > 28 days and 32 infants with missing data. The association between serum phosphate level and hospital Los. remained reliable (supplemental table S6).

## Discussion

To the best of our knowledge, this is the first study to consistently observe a significant negative association between serum phosphate levels and hospital Los. in infants with neonatal sepsis. The hospital Los. was prolonged by 7.53 days with every 1 mmol/L decrease in serum phosphate levels, and further exploratory subgroup analysis revealed no significant interactions. Therefore, this association is reliable and independent of essential covariates and confounders.

Critically ill patients are prone to electrolyte and mineral metabolic disorders, including changes in serum phosphate levels [16]. Patients with sepsis can develop hypophosphatemia and hyperphosphatemia [17]. Hypophosphatemia in adults is defined as a serum phosphate < 0.8 mmol/L [18]. Its prevalence in the ICU is 15.4%–85% and approximately 60% in the PICU [19–21]. Neonates have high levels of phosphorus in their blood to maintain their growth and developmental requirements, and hypophosphatemia is typically defined as serum phosphate < 2.0 mmol/L [22]. However, there are few studies on phosphate metabolism disorder in neonatal sepsis. Our study showed that the prevalence of hypophosphatemia in neonatal patients with sepsis was 60%, which is similar to its prevalence in patients in the PICU, and slightly lower than the previously reported prevalence of hypophosphatemia in neonates with sepsis [9]. Hyperphosphatemia is defined as serum phosphate ≥ 1.45 mmol/L, with an incidence of 5.6%–45% in critically ill patients [23]. However, in neonates, it is defined as serum phosphate >2.6 mmol/L. The incidence of hyperphosphatemia in our study was 15.8%, which is lower than that reported in another neonatal sepsis study (26.0%) [24]. This difference in results may be related to the difference in the time point of testing selected for serum phosphate in the previous study, which selected serum phosphate prior to antibiotic application; however, our study selected serum phosphate within 72 h of the diagnosis of sepsis.

Comparison of baseline characteristics between the serum phosphate tertiles groups revealed longer hospital Los.and ICU Los., and lower ionized calcium levels in the low phosphate level group. The lower serum phosphate levels and relatively higher ionized calcium levels in infants with neonatal sepsis are consistent with those of previous studies [24], which have been linked to bacteremia and the effects of inflammatory mediators on parathyroid hormone secretion and function [25]. Our study showed that infants in the high-phosphate group had shorter days of age, a lower percentage of late-onset sepsis, and higher hemoglobin concentration, which were associated with a gradual decrease in neonatal serum phosphate levels with days of age [8]. In the high phosphate level group, there was a higher percentage of AKI and creatinine and lactate concentrations than in the other two groups, because hyperphosphatemia in patients with sepsis is associated with an inflammatory response leading to apoptosis, destruction, and renal impairment [26], as well as tissue ischemia, hypoxia, and hyperlactatemia [27, 28].

Hypophosphatemia in patients with sepsis is caused by the redistribution of phosphate and renal loss. Urinary loss of inorganic phosphate is significantly higher in neonates with sepsis than in those with respiratory distress syndrome [9]. Hypophosphatemia inhibits neutrophil function and increases bacterial virulence; therefore, sepsis and hypophosphatemia are mutually exacerbated. Acute hypophosphatemia can affect different organs and cause respiratory dysfunction, myocardial contractile weakness, arrhythmia, leukocyte dysfunction, and insulin resistance [29]. However, the relationship between hypophosphatemia and prognosis in patients with sepsis remains controversial. Some studies have shown that hypophosphatemia is associated with 28-day and 90-day mortality and longer hospital Los. and mechanical ventilation for patients [7, 30]. However, a recent meta-analysis indicated that sepsis patients with low serum phosphate levels before interventions may have a reduced risk of all-cause mortality, shorter ICU Los., and hospital Los.; however, the results were not statistically significant, and the variation in the predictive outcomes for sepsis patients with low phosphate levels could be attributed to the disparate thresholds for what is considered low phosphate levels and the timing of blood tests across the referenced studies [6].

This study is the first to report that hypophosphatemia is associated with prolonged hospital Los. in neonates with sepsis. After adjusting for all potential covariates, the results remained stable, consistent with those of studies in critically ill children [31]. Smooth curve fitting to assess the linear relationship between serum phosphate levels and hospital Los. showed a linear negative correlation. To enhance the reliability of our results, we conducted subgroup analyses based on gender, premature, LOS, severe sepsis, acute renal failure, and hypocalcemia. Our findings revealed that lower serum phosphate levels was consistently associated with longer hospital Los. across all subgroups. Because the age of preterm infants included in this study was ≤ 90 days, and the age of full-term infants was ≤ 28 days, in order to discharge the possible influence of the difference in the age of inclusion on the results, sensitivity analyses were performed with the 14 preterm infants who were more than 28 days old removed and the results remained stable (S6). We excluded 32 patients with missing data from the sensitivity analysis, and the results remained consistent (S6).

Most studies have shown that patients with sepsis and high serum phosphate levels have a significantly increased all-cause mortality risk [6, 32]. A predictive modeling study on the risk of neonatal sepsis death showed that hypocalcemia and hyperphosphatemia were associated with an increased risk of death in neonates with sepsis [24], but their study did not examine the occurrence of hypophosphatemia in neonates with sepsis and the relationship between serum phosphate levels and hospital Los. In our study; the correlation between serum phosphate levels and ICU Los. in neonates with sepsis became insignificant after adjusting for laboratory indicators. In addition, no correlation was found between serum phosphate levels and mortality or the severity of sepsis in neonates, which may be related to the relatively small sample size.

This study has several limitations. First, because of the single-center retrospective study design, despite our diligent attempts to account for possible confounding elements and perform subgroup analyses, the presence of selection and confounding bias remains an unavoidable constraint. Second, the sample size in our study was relatively small, which might have influenced the accuracy of our results. Only in-hospital deaths are recorded in the database. Due to the small number, it may affect the research results on the association between serum phosphorus levels and in-hospital mortality in neonates with sepsis.Due to the small sample size, there was no significant difference in baseline characteristics of infants with hypophosphatemia stratified by severity. Although the results of the multivariate linear regression analysis did not show statistical differences, the effect values showed a negative correlation between serum phosphate levels and hospital Los.(S10).Third, because the medication information in the PIC database was incomplete, we could not conduct an deep analysis of whether phosphate supplementation influenced clinical outcomes.Among the 120 infants with neonatal sepsis in this study, 67 infants had medication records and were stratified into three groups based on phosphorus supplementation status. Nine infants did not receive phosphorus supplementation, 17 infants received sodium glycero-phosphate supplementation before sepsis and continued to do so after sepsis, and 41 patients received sodium glycero-phosphate supplementation after sepsis. Baseline comparisons among these three groups revealed differences in serum phosphate levels during sepsis. Infants who received phosphorus supplementation before sepsis had the lowest serum phosphate levels and longer hospital Los. (*p* < 0.05), as shown in Table S7. Multiple linear regression analysis was then performed to examine the association between serum phosphate levels and hospital Los. in these 67 infants. After adjusting for the confounding factor of pre-sepsis phosphorus supplementation, serum phosphate levels remained negatively correlated with hospital Los. (β, −8.08; 95% CI, −15.45~−0.71; *P* = 0.035), as shown in Table S8. However, due to retrospective study and small sample size, there were differences in baseline serum phosphate levels among the three groups of infants. So we can not draw a definitive conclusion regrading the prognostic impact of phosphorus supplementation. Future studies will focus on random multicenter, large sample size, and control trial studies to confirm the causal relationship between serum phosphate levels and outcomes of neonatal sepsis.

## Conclusion

To summarize, hypophosphatemia is highly prevalent among neonates with sepsis. Lower serum phosphate levels are associated with prolonged hospital Los. and may affect the prognosis of neonatal sepsis. Strengthening the monitoring of serum phosphate levels in neonates with sepsis and maintaining the homeostasis of phosphate in the body can promote refined management of neonatal sepsis and help improve the prognosis of the patients.

## Supplementary Information

Supplementary Material 1.

## Data Availability

All data in the article can be obtained from the PIC database (https://www.physionet.org/content/picdb/1.0.0/).

## Abbreviations

PIC: Pediatric Intensive Care
hospital Los.: Length of hospital stay
ICU: Intensive Care Unit
ICU Los.: Length of ICU stay
LOS: Late-onset sepsis
NEC: Necrotizing enterocolitis
CHD: Congenital heart disease
ICH: Intracranial hemorrhage
AKI: Acute kidney injury
ALI: Acute liver injury
iCa: Ionized calcium
ALT: Alanine aminotransferase
BUN: Blood urea nitrogen
CR: Creatinine
CRP: C-reactive protein
LAC: Lactate
