# Supplementary material for: Association between serum phosphate levels and length of hospital stay in infants with neonatal sepsis: a retrospective cohort study
Source: BMC Pediatr. 2025 Oct 21;25:827. doi: 10.1186/s12887-025-06209-z (PMC12539105; doi:10.1186/s12887-025-06209-z)
Supplement: Supplementary file 1 — Supplementary Material 1. [file 12887_2025_6209_MOESM1_ESM.docx]

S1: Baseline characteristics of participants

| **Co-variate** | **Total**  (n = 120) | **Phosphate level(mmol/L)（mg/dl）** | | | **P -value** |
| --- | --- | --- | --- | --- | --- |
|  |  | <2.0(6.2)  (n = 72) | 2.0(6.2) ≤pi≤2.6(8.06)  (n = 29) | >2.6(8.06)  (n = 19) |  |
| **Baseline characteristics** |  |  |  |  |  |
| age,days | 3.0 (1.0, 17.0) | 6.0 (2.0, 23.5) | 2.0 (0.0, 14.0) | 2.0 (1.0, 5.0) | 0.009 |
| gender, Males,n (%) | 75 (62.5) | 45 (62.5) | 18 (62.1) | 12 (63.2) | 0.997 |
| ICU weight, kg | 2.5 ± 0.9 | 2.3 ± 0.9 | 2.5± 1.0 | 2.9 ±0.6 | 0.102 |
| premature, n (%) | 53 (44.2) | 36 (50) | 13 (44.8) | 4 (21.1) | 0.077 |
| LOS, n (%) | 73 (60.8) | 52 (72.2) | 14 (48.3) | 7 (36.8) | 0.005 |
| **Outcome** |  |  |  |  |  |
| In-hospital Mortality, n (%) | 10 ( 8.3) | 4 (5.6) | 2 (6.9) | 4 (21.1) | 0.122 |
| severe.sepsis, n (%) | 34 (28.3) | 23 (31.9) | 4 (13.8) | 7 (36.8) | 0.125 |
| hospital.Los, days | 28.9 ± 21.7 | 33.7 ± 22.5 | 26.7 ± 20.5 | 14.1 ± 11.1 | 0.001 |
| ICU Los, days | 24.8 ± 21.3 | 29.7 ± 23.8 | 19.9 ± 15.9 | 13.9 ± 11.1 | 0.005 |
| **Commodities** |  |  |  |  |  |
| bacterial meningitis, n (%) | 13 (10.8) | 6(8.3) | 5 (17.2) | 2 (10.5) | 0.429 |
| digestive tract anomaly, n (%) | 13 (10.8) | 9 (12.5) | 3 (10.3) | 1 (5.3) | 0.837 |
| NEC and peritonitis, n (%) | 11 ( 9.2) | 9 (12.5) | 1(3.4) | 1 (5.3) | 0.397 |
| CHD, n (%) | 15 (12.5) | 10(13.9) | 3(10.3) | 2 (10.5) | 0.92 |
| ICH, n (%), | 21 (17.5) | 16(22.2) | 4( 13.8) | 1(5.3) | 0.225 |
| neonatal asphyxia,n (%) | 9(7.5) | 4 (5.6) | 3 (10.3) | 2(10.5) | 0.547 |
| respiratory failure, n (%) | 23(19.2) | 19 (33.3) | 2 (10.3) | 2(21.1) | 0.06 |
| AKI, n (%) | 23/120 (19.2) | 9 /72(12.5) | 6/29 (20.7) | 8 /19(42.1) | 0.02 |
| ALI, n (%) | 11 /120( 9.2) | 4/72 (5.6) | 4/29 (13.8) | 3 /19(15.8) | 0.204 |
| **Laboratory examination** |  |  |  |  |  |
| blood culture, positive,n (%) | 47 (39.2) | 31 (43.1) | 8 (27.6) | 8 (42.1) | 0.34 |
| phosphate, mmol/L | 1.9 ± 0.7 | 1.5 ± 0.3 | 2.2 ± 0.1 | 3.1 ± 0.5 | < 0.001 |
| iCa, mmol/L | 1.1 ± 0.2 | 1.1 ± 0.2 | 1.1 ± 0.2 | 1.1 ± 0.2 | 0.119 |
| Total Ca,mmol/L | 2.1 ± 0.6 | 2.1 ± 0.8 | 2.1 ± 0.3 | 2.1 ± 0.3 | 0.982 |
| ALT, U/L | 14.0 (8.0, 28.2) | 13.0 (8.0, 32.0) | 11.0 (6.0, 23.0) | 16.0 (13.0, 27.5) | 0.232 |
| BUN, mmol/L | 5.8 ± 4.0 | 5.3 ± 3.6 | 6.6 ± 4.8 | 6.5 ± 3.9 | 0.266 |
| CR, µmol/L | 78.3 ± 40.5 | 67.8 ± 32.1 | 91.2 ± 51.9 | 98.5 ± 38.2 | 0.002 |
| CRP,mg/L | 52.6 ± 49.5 | 61.0 ± 54.3 | 36.8 ±36.2 | 43.4 ± 40.8 | 0.064 |
| WBC, ×10^9^/L | 12.6 ± 10.2 | 12.8 ± 11.2 | 12.2 ± 7.8 | 12.1 ± 9.5 | 0.929 |
| platelets, ×10^9^/L | 177.0 ± 140.5 | 162.4 ± 123.8 | 208.8 ± 178.7 | 183.6 ± 133.6 | 0.318 |
| Hemoglobin,g/L | 131.9± 319 | 125.8 ± 29.5 | 139.2 ± 30.0 | 144.2 ± 38.9 | 0.030 |
| PH | 7.3 ± 0.3 | 7.3 ± 0.3 | 7.3 ± 0.2 | 7.3 ± 0.2 | 0.764 |
| LAC,mmol/L | 2.3(1.5,4.4) | 2.1(1.4,3.3) | 2.0(1.8,2.7) | 4.5(3.4,8.8) | < 0.001 |

Abbreviations:ICU weight:first weight in ICU, hospital.Los:length of hospital stay,ICU Los:length of hospital stay,LOS:Late onset neonatal sepsis,NEC:necrotizing enterocolitis in newborns,CHD:congenital heart disease;ICH:intracranial hemorrhage;AKI:acute renal injury; ALI:acute liver function injury , ALT: alanine aminotransferase, BUN:blood urea nitrogen, CRP:C reactive protein,;PH:pH value in blood gas ,LAC:lactic acid

S2 Association of covariates and hospital Mortality

| **Variable** | **OR_95CI** | **P_value** |
| --- | --- | --- |
| Age(days) | 0.99 (0.95~1.04) | 0.742 |
| Gender,n (%): Males | 1.44 (0.35~5.88) | 0.61 |
| ICU weight | 0.28 (0.04~1.74) | 0.172 |
| Premature,n (%) | 0.83 (0.22~3.11) | 0.782 |
| LONS,n (%) | 0.62 (0.17~2.26) | 0.467 |
| Severe sepsis | 356056428.88 (0~Inf) | 0.992 |
| Hospital.Los | 0.73 (0.61~0.88) | 0.001 |
| ICU.Los. | 0.8 (0.68~0.94) | 0.006 |
| Bacterial meningitis,n (%) | 0 (0~Inf) | 0.993 |
| Digestive tract anomaly,n (%) | 0 (0~Inf) | 0.993 |
| CHD,n (%) | 0 (0~Inf) | 0.992 |
| NEC and peritonitis,n (%) | 1.11 (0.13~9.69) | 0.924 |
| ICH,n (%) | 0 (0~Inf) | 0.991 |
| Neonatal asphyxia,n(%) | 0 (0~Inf) | 0.994 |
| Respiratory failure,n(%) | 1.93 (0.46~8.11) | 0.37 |
| ALI,n(%) | 9.81 (2.24~43.04) | 0.002 |
| AKI ,n (%) | 1.93 (0.46~8.11) | 0.37 |
| Blood culture,positive,n (%) | 4.08 (1~16.68) | 0.05 |
| Phosphate,mmol/L | 1.98 (0.86~4.55) | 0.106 |
| iCa,mmol/L | 0.2 (0~11.89) | 0.437 |
| Total Ca, mmol/L | 0.24 (0.03~1.67) | 0.149 |
| CRP,mg/L | 1 (0.98~1.01) | 0.803 |
| WBC, ×10^9^ /L | 1.01 (0.94~1.07) | 0.824 |
| Hemoglobin, g/L | 1 (0.98~1.02) | 0.837 |
| Platelets, ×10^9^ /L | 0.99 (0.99~1) | 0.133 |
| ALT,U/L | 1 (1~1.01) | 0.31 |
| BUN ,mmol/L | 1.08 (0.94~1.24) | 0.282 |
| CR,μmol/L | 1.01 (0.99~1.02) | 0.293 |
| PH | 0.25 (0.05~1.32) | 0.103 |
| LAC,mmol/L | 1.21 (1.08~1.35) | 0.001 |

Abbreviations: ICU weight:first weight in ICU, hospital.Los:length of hospital stay,ICU Los:length of hospital stay,LOS:Late onset neonatal sepsis,NEC:necrotizing enterocolitis in newborns,CHD:congenital heart disease;ICH:intracranial hemorrhage;AKI:acute renal injury; ALI:acute liver function injury , ALT: alanine aminotransferase, BUN:blood urea nitrogen, CRP:C reactive protein,;PH:pH value in blood gas ,LAC:lactic acid

S3 Association of covariates and severe sepsis

| **Variable** | **OR_95CI** | **P_value** |
| --- | --- | --- |
| Age(days) | 0.99 (0.96~1.02) | 0.366 |
| Gender,n (%): Males | 1.14 (0.5~2.61) | 0.754 |
| ICU weight | 1.29 (0.77~2.18) | 0.332 |
| Premature,n (%) | 1 (0.45~2.22) | 0.995 |
| LOS,n (%) | 0.38 (0.17~0.86) | 0.02 |
| Hospital Mortality | 152452244.23 (0~Inf) | 0.988 |
| Hospital.Los | 0.96 (0.94~0.99) | 0.003 |
| ICU.Los | 0.97 (0.95~1) | 0.026 |
| Bacterial meningitis,n (%) | 1.68 (0.51~5.56) | 0.395 |
| Digestive tract anomaly,n (%) | 1.14 (0.33~3.99) | 0.837 |
| CHD,n (%) | 4.8 (1.56~14.81) | 0.006 |
| NEC and peritonitis,n (%) | 2.3 (0.65~8.11) | 0.196 |
| ICH,n (%) | 6.04 (2.21~16.47) | <0.001 |
| Neonatal asphyxia,n(%) | 0.71 (0.14~3.58) | 0.674 |
| Respiratory failure,n(%) | 3.72 (1.44~9.58) | 0.007 |
| ALI,n(%) | 5.31 (1.44~19.56) | 0.012 |
| AKI ,n (%) | 3.72 (1.44~9.58) | 0.007 |
| Blood culture,positive,n (%) | 2.21 (0.99~4.96) | 0.054 |
| Phosphate,mmol/L | 1.19 (0.66~2.14) | 0.561 |
| iCa,mmol/L | 0 (0~0.08) | <0.001 |
| Total Ca, mmol/L | 0.07 (0.02~0.28) | <0.001 |
| CRP,mg/L | 1 (0.99~1.01) | 0.886 |
| WBC, ×10^9^ /L | 0.98 (0.94~1.02) | 0.372 |
| Hemoglobin, g/L | 1 (0.99~1.02) | 0.62 |
| Platelets, ×10^9^ /L | 0.99 (0.99~1) | 0.01 |
| ALT,U/L | 1.01 (1~1.02) | 0.026 |
| BUN ,mmol/L | 1.13 (1.02~1.25) | 0.015 |
| CR,μmol/L | 1.01 (1~1.02) | 0.01 |
| PH | 0.19 (0.02~1.47) | 0.113 |
| LAC,mmol/L | 1.27 (1.11~1.44) | <0.001 |

S4 Multi-variable logistic regression analyses of phosphate level and mortality and severe sepsis

| **Variable** | **Hospital mortality** | | | | **Severe sepsis** | | | |
| --- | --- | --- | --- | --- | --- | --- | --- | --- |
|  | **Model I** | | **Model II** | | **Model I** | | **Model II** | |
|  | **OR (95%CI)** | **P** | **OR (95%CI)** | **P** | **OR (95%CI)** | **P** | **OR (95%CI)** | **P** |
| Phosphate mmol/L  (mg/dL） | 1.98 (0.86~4.55) | 0.106 | 2.03 (0.84~4.94) | 0.116 | 1.19 (0.66~2.14) | 0.561 | 1.05(0.57~1.96) | 0.872 |
| Phosphate tertiles |  |  |  |  |  |  |  |  |
| Low(0.55-1.60)  (1.71-4.96) | Reference |  | Reference |  | Reference |  | Reference |  |
| Mild(1.61-2.12)  (4.99-6.57) | 1(0.13~7.47) | 1 | 0.93 (0.12~7.02) | 0.941 | 1.44 (0.55~3.83) | 0.46 | 1.46(0.54~3.97) | 0.456 |
| High(2.13-4.93)  (6.6-15.28) | 3.35 (0.63~17.74) | 0.155 | 3.54(0.61~20.51) | 0.158 | 1.14 (0.42~3.08) | 0.799 | 0.91(0.32~2.58) | 0.857 |

Model I: no other covariates were adjusted.ModelⅡ: we adjusted age, gender,ICU weight.

S5 Subgroup analyses of phosphate level and the length of hospital stay

| **Subgroup** | **Variable** | **n** | **crude.β_95CI** | **crude.P_value** | **adj.β_95CI** | **adj.P_value** | **P.for.interaction** |
| --- | --- | --- | --- | --- | --- | --- | --- |
| gender |  |  |  |  |  |  |  |
| female | phosphate | 45 | -13.02 (-20.42~-5.61) | 0.001 | -7.12 (-15.48~1.24) | 0.104 | 0.543 |
| male | phosphate | 75 | -11.54 (-19.33~-3.75) | 0.005 | -3.87 (-11.6~3.86) | 0.33 |  |
| premature |  |  |  |  |  |  |  |
| no | phosphate | 67 | -6.93 (-11.32~-2.54) | 0.003 | -5.84 (-10.25~-1.43) | 0.012 | 0.1 |
| yes | phosphate | 53 | -16.32 (-28.12~-4.52) | 0.009 | -8.72 (-21.04~3.6) | 0.173 |  |
| LOS |  |  |  |  |  |  |  |
| no | phosphate | 47 | -7.99 (-13.95~-2.03) | 0.012 | -8.23 (-13.17~-3.29) | 0.002 | 0.9 |
| yes | phosphate | 73 | -12.84 (-21.99~-3.69) | 0.008 | -2.59 (-12.73~7.56) | 0.619 |  |
| severe.sepsis |  |  |  |  |  |  |  |
| no | phosphate | 86 | -15.46 (-22.65~-8.27) | <0.001 | -7.43 (-14.49~-0.37) | 0.043 | 0.123 |
| yes | phosphate | 34 | -6.72 (-13.76~0.32) | 0.07 | -4.48 (-12.26~3.31) | 0.273 |  |
| AKI |  |  |  |  |  |  |  |
| no | phosphate | 97 | -12.18 (-19.22~-5.14) | 0.001 | -4.87 (-11.71~1.97) | 0.167 | 0.991 |
| yes | phosphate | 23 | -11.69 (-20.14~-3.24) | 0.013 | -6.27 (-21.48~8.95) | 0.438 |  |
| iCa group |  |  |  |  |  |  |  |
| <1.0mmol/L | phosphate | 24 | -7.45 (-15.54~0.63) | 0.085 | -4.38 (-17.22~8.47) | 0.518 | 0.477 |
| ≥1.0mmol/L | phosphate | 96 | -13.63 (-20.63~-6.64) | <0.001 | -6.18 (-12.72~0.37) | 0.068 |  |

S6 Sensitivity analysis of phosphate level and the length of hospital stay

| **patients** | **Variable** |  | **Model I** |  | **Model II** |  |
| --- | --- | --- | --- | --- | --- | --- |
|  |  | **n** | **β (95%CI)** | **P value** | **β (95%CI)** | **P value** |
| age≤28 days | Phosphate mmol/L | 106 | -11.23 (-16.95~-5.51) | <0.001 | -6.98 (-12.3~-1.67) | 0.012 |
| No missing date | Phosphate mmol/L | 88 | -13.25 (-19.69~-6.8) | <0.001 | -9(-15.27~-2.73) | 0.006 |

Model I : no other covariates were adjusted. Model Ⅱ: we adjusted age ,gender.,ICU weight, premature,LOS, hospital mortality,severe sepsis,CHD,respiratory failure,, CRP,iCa,ALT,LAC,hemoglobin,CR.

S7 Baseline characteristics of participants received phosphate supplementation

| **Co-variate** | **Total (n = 67)** | **phosphate supplementation** | | | **P -value** |
| --- | --- | --- | --- | --- | --- |
|  |  | No(n = 9) | After(n = 41) | Before(n = 17) |  |
| **Baseline characteristics** |  |  |  |  |  |
| age,days | 3.0 (1.0, 18.5) | 6.0 (2.0, 10.0) | 2.0 (1.0, 17.0) | 4.0 (0.0, 25.0) | 0.729 |
| gender, Males,n (%) | 44 (65.7) | 4 (44.4) | 29 (70.7) | 11 (64.7) | 0.347 |
| ICUweight, kg | 2.5 ± 0.9 | 2.9 ± 1.1 | 2.6 ± 0.9 | 2.1 ± 0.5 | 0.056 |
| premature, n (%) | 37 (55.2) | 5 (55.6) | 17 (41.5) | 15 (88.2) | 0.005 |
| LOS, n (%) | 41 (61.2) | 6 (66.7) | 19 (46.3) | 16 (94.1) | 0.002 |
| **Outcome** |  |  |  |  |  |
| In-hospital Mortality, n (%) | 1 ( 1.5) | 0 (0) | 1 (2.4) | 0 (0) | 1 |
| severe.sepsis, n (%) | 26 (38.8) | 3 (33.3) | 19 (46.3) | 4 (23.5) | 0.243 |
| hospital.los, days | 31.8 ± 20.3 | 23.2 ± 18.6 | 29.0 ± 17.6 | 43.2 ± 23.6 | 0.018 |
| ICU Los, days | 21.7 (5.1, 40.3) | 1.5 (1.0, 3.2) | 21.7 (11.0, 39.9) | 32.1 (12.0, 42.8) | 0.003 |
| **Commodities** |  |  |  |  |  |
| bacterial meningitis, n (%) | 11 (16.4) | 2 (22.2) | 8 (19.5) | 1 (5.9) | 0.453 |
| digestive tract anomaly, n (%) | 12 (17.9) | 2 (22.2) | 5 (12.2) | 5 (29.4) | 0.24 |
| NEC and peritonitis, n (%) | 11 (16.4) | 2 (22.2) | 3 (7.3) | 6 (35.3) | 0.023 |
| CHD, n (%) | 15 (22.4) | 1 (11.1) | 7 (17.1) | 7 (41.2) | 0.097 |
| ICH, n (%), | 21 (31.3) | 2 (22.2) | 15 (36.6) | 4 (23.5) | 0.601 |
| neonatal asphyxia,n (%) | 9 (13.4) | 1 (11.1) | 6 (14.6) | 2 (11.8) | 1 |
| respiratory failure, n (%) | 14 (20.9) | 1 (11.1) | 8 (19.5) | 5 (29.4) | 0.56 |
| AKI, n (%) | 13 (19.4) | 3 (33.3) | 9 (22) | 1 (5.9) | 0.203 |
| ALI, n (%) | 7 (10.4) | 1 (11.1) | 4 (9.8) | 2 (11.8) | 1 |
| **Laboratory examination** |  |  |  |  |  |
| blood culture, positive,n (%) | 26 (38.8) | 5 (55.6) | 14 (34.1) | 7 (41.2) | 0.445 |
| phosphate, mmol/L | 1.9 ± 0.6 | 2.5 ± 1.0 | 1.9 ± 0.5 | 1.7 ± 0.5 | 0.002 |
| iCa, mmol/L | 1.1 ± 0.2 | 1.1 ± 0.1 | 1.1 ± 0.2 | 1.1 ± 0.2 | 0.379 |
| Total Ca,mmol/L | 2.0 ± 0.3 | 2.2 ± 0.3 | 2.0 ± 0.3 | 2.2 ± 0.2 | 0.041 |
| ALT, U/L | 14.0 (8.0, 29.5) | 13.0 (10.0, 23.0) | 14.0 (8.0, 27.0) | 11.0 (8.0, 54.0) | 0.978 |
| BUN, mmol/L | 4.8 (3.2, 7.4) | 6.8 (3.2, 8.4) | 5.5 (3.4, 7.7) | 4.6 (2.9, 5.5) | 0.54 |
| CR, µmol/L | 75.2 ± 32.0 | 79.1 ± 26.6 | 80.8 ± 34.5 | 59.6 ± 23.6 | 0.066 |
| CRP,mg/L | 36.0 (16.3, 81.3) | 16.6 (15.8, 18.8) | 46.8 (20.6, 82.5) | 32.4 (8.5, 98.0) | 0.15 |
| WBC, ×10^9^/L | 8.3 (4.8, 18.1) | 18.2 (10.7, 19.9) | 6.1 (4.5, 13.9) | 9.3 (5.8, 19.4) | 0.096 |
| platelets, ×10^9^/L | 148.0 (55.5, 246.0) | 224.0 (115.0, 284.0) | 130.0 (49.0, 200.0) | 230.0 (131.0, 360.0) | 0.024 |
| Hemoglobin,g/L | 132.2 ± 31.3 | 134.4 ± 22.3 | 134.1 ± 33.6 | 126.4 ± 30.4 | 0.681 |
| PH | 7.3 ± 0.2 | 7.3 ± 0.2 | 7.3 ± 0.2 | 7.3 ± 0.2 | 0.919 |
| LAC,mmol/L | 2.2 (1.4, 5.2) | 2.8 (1.4, 3.6) | 2.5 (1.8, 7.3) | 1.4 (1.2, 2.3) | 0.014 |

No: no received phosphate supplementation; After:received sodium glycero-phosphate supplementation after sepsis; Before:received sodium glycero-phosphate supplementation before sepsis and continued to do so after sepsis.

S8 Multi-variable linear regression analyses of phosphate level and the length of hospital stay in participants received phosphate supplementation

| **Variable** |  | **Model I** |  | **Model II** |  |
| --- | --- | --- | --- | --- | --- |
|  | **n** | **β (95%CI)** | **P value** | **β (95%CI)** | **P value** |
| Phosphate mmol/L | 67 | -10.25 (-17.59~-2.91) | 0.008 | -8.08 (-15.45~-0.71) | 0.035 |

Model I : no other covariates were adjusted. Model Ⅱ: we adjusted pre-sepsis phosphorus supplementation.

S9 Baseline characteristics of participants with hypophosphatemia

| **Co-variate** | **Total (n = 72)** | **Phosphate level(mmol/L)（mg/dL）** | | | **P -value** |
| --- | --- | --- | --- | --- | --- |
|  |  | severe  0.55-1.44(1.71-4.46)  (n = 24) | moderate  1.45-1.69(4.50-5.24)  (n = 22) | mild  1.71-1.96(5.30-6.08)  (n = 26) |  |
| **Baseline characteristics** |  |  |  |  |  |
| age, days | 6.0 (2.0, 23.5) | 6.0 (2.0, 29.5) | 4.5 (1.2, 10.8) | 7.5 (2.2, 22.2) | 0.54 |
| gender, Males,n (%) | 45 (62.5) | 15 (62.5) | 13 (59.1) | 17 (65.4) | 0.904 |
| ICUweight, kg | 2.3 ± 0.9 | 2.2 ± 0.9 | 2.6 ± 0.9 | 2.3 ± 0.8 | 0.513 |
| premature, n (%) | 36 (50.0) | 12 (50) | 10 (45.5) | 14 (53.8) | 0.845 |
| LOS, n (%) | 52 (72.2) | 18 (75) | 13 (59.1) | 21 (80.8) | 0.231 |
| **Outcome** |  |  |  |  |  |
| In-hospital Mortality, n (%) | 4 ( 5.6) | 2 (8.3) | 1 (4.5) | 1 (3.8) | 0.831 |
| severe.sepsis, n (%) | 23 (31.9) | 7 (29.2) | 5 (22.7) | 11 (42.3) | 0.328 |
| hospital.Los, days | 29.0 (18.5, 45.8) | 35.0 (21.2, 51.8) | 24.0 (17.5, 41.5) | 28.0 (15.2, 42.8) | 0.35 |
| ICU Los, days | 24.3 (11.5, 42.8) | 29.4 (13.9, 45.5) | 21.2 (9.9, 34.7) | 28.0 (6.5, 41.5) | 0.199 |
| **Comorbidities** |  |  |  |  |  |
| bacterial meningitis, n (%) | 6 ( 8.3) | 2 (8.3) | 3 (13.6) | 1 (3.8) | 0.431 |
| digestive tract anomaly, n (%) | 9 (12.5) | 3 (12.5) | 2 (9.1) | 4 (15.4) | 0.912 |
| NEC and peritonitis, n (%) | 9 (12.5) | 2 (8.3) | 3 (13.6) | 4 (15.4) | 0.739 |
| CHD, n (%) | 10 (13.9) | 3 (12.5) | 3 (13.6) | 4 (15.4) | 1 |
| ICH, n (%), | 16 (22.2) | 4 (16.7) | 5 (22.7) | 7 (26.9) | 0.728 |
| neonatal asphyxia,n (%) | 4 ( 5.6) | 2 (8.3) | 1 (4.5) | 1 (3.8) | 0.852 |
| respiratory failure, n (%) | 19 (26.4) | 6 (25) | 3 (13.6) | 10 (38.5) | 0.148 |
| AKI, n (%) | 9 (12.5) | 2 (8.3) | 4 (18.2) | 3 (11.5) | 0.602 |
| ALI, n (%) | 4 ( 5.6) | 1 (4.2) | 1 (4.5) | 2 (7.7) | 1 |
| **Laboratory examination** |  |  |  |  |  |
| blood culture, positive,n (%) | 31 (43.1) | 13 (54.2) | 9 (40.9) | 9 (34.6) | 0.367 |
| phosphate, mmol/L | 1.5 ± 0.3 | 1.2 ± 0.2 | 1.6 ± 0.1 | 1.8 ± 0.1 | < 0.001 |
| iCa, mmol/L | 1.1 ± 0.2 | 1.2 ± 0.2 | 1.1 ± 0.1 | 1.1 ± 0.2 | 0.177 |
| Total Ca,mmol/L | 2.1 ± 0.8 | 2.3 ± 1.3 | 2.0 ± 0.3 | 2.1 ± 0.3 | 0.531 |
| ALT, U/L | 13.0 (8.0, 32.0) | 15.5 (8.0, 38.5) | 14.5 (11.0, 24.0) | 10.5 (7.2, 18.2) | 0.338 |
| BUN, mmol/L | 4.5 (2.8, 6.4) | 4.5 (3.2, 6.2) | 5.6 (3.1, 8.0) | 3.5 (2.6, 5.6) | 0.334 |
| CR, µmol/L | 67.8 ± 32.1 | 70.7 ± 38.8 | 68.6 ± 23.3 | 64.5 ± 32.6 | 0.791 |
| CRP,mg/L | 42.7 (16.9, 102.1) | 52.4 (14.0, 116.6) | 21.8 (17.0, 116.3) | 42.7 (20.5, 73.3) | 0.927 |
| WBC, ×10^9^/L | 9.2 (4.8, 17.7) | 9.3 (5.7, 17.7) | 11.9 (5.2, 19.8) | 6.8 (3.8, 13.8) | 0.48 |
| platelets, ×10^9^/L | 142.0 (51.5, 229.2) | 135.5 (58.5, 202.8) | 108.0 (32.2, 205.5) | 184.0 (129.2, 287.8) | 0.091 |
| Hemoglobin,g/L | 125.8 ± 29.5 | 128.1 ± 25.9 | 118.6 ± 36.8 | 129.8 ± 25.3 | 0.384 |
| PH | 7.3 ± 0.3 | 7.3 ± 0.1 | 7.4 ± 0.1 | 7.2 ± 0.5 | 0.201 |
| LAC,mmol/L | 2.1 (1.4, 3.3) | 2.2 (1.5, 2.6) | 2.4 (1.5, 3.6) | 2.1 (1.3, 4.0) | 0.835 |

S10 Multi-variable linear regression analyses of phosphate level and the length of hospital stay in participants

with hypophosphatemia

| **Variable** | **Total**  **n** | **Hospital Los.** |  |  |  |
| --- | --- | --- | --- | --- | --- |
|  |  | **Model I** |  | **Model II** |  |
|  |  | **OR (95%CI)** | **P** | **OR (95%CI)** | **P** |
| Phosphate mmol/L  (mg/dL) | 72 | -13.72 (-30.58~3.14) | 0115 | -13.8 (-29.27~1.67) | 0.085 |
| Phosphate tertiles |  |  |  |  |  |
| Severe (0.55-1.61)  (1.71-4.46) | 24 | Reference |  | Reference |  |
| Moderate (1.45-1.69)  (4.50-5.24) | 22 | -10.02 (-22.95~2.92) | 0.134 | -9.73 (-21.61~2.16) | 0.114 |
| Mild (1.71-1.96)  (5.30-6.08) | 26 | -8.48 (-20.89~3.92) | 0.185 | -9.18 (-20.54~2.19) | 0.118 |

Model I : no other covariates were adjusted. Model Ⅱ: we adjusted age ,gender, premature.
